# Perspectives of using Illumina MiSeq for identification of arbuscular mycorrhizal fungi

**DOI:** 10.18699/VJ19.38-o

**Published:** 2020-03

**Authors:** A.A. Kryukov, A.O. Gorbunova, E.M. Machs, Y.V. Mikhaylova, A.V. Rodionov, P.M. Zhurbenko, A.P. Yurkov

**Affiliations:** All-Russian Research Institute for Agricultural Microbiology, St. Petersburg, Russia; All-Russian Research Institute for Agricultural Microbiology, St. Petersburg, Russia Saint Petersburg State University, Biological Faculty, St. Petersburg, Russia; Komarov Botanical Institute of the Russian Academy of Sciences, St. Petersburg, Russia; Komarov Botanical Institute of the Russian Academy of Sciences, St. Petersburg, Russia; Saint Petersburg State University, Biological Faculty, St. Petersburg, Russia Komarov Botanical Institute of the Russian Academy of Sciences, St. Petersburg, Russia; Komarov Botanical Institute of the Russian Academy of Sciences, St. Petersburg, Russia; All-Russian Research Institute for Agricultural Microbiology, St. Petersburg, Russia

**Keywords:** Glomeromycotina, arbuscular mycorrhiza, Illumina MiSeq, Rhizophagus irregularis, R. invermaius, Paraglomus laccatum, Claroideoglomus etunicatum, Acaulospora, Glomeromycotina, арбускулярная микориза, Illumina MiSeq, Rhizophagus irregularis, R. invermaius, Paraglomus laccatum, Claroideoglomus etunicatum, Acaulospora

## Abstract

Arbuscular mycorrhiza fungi (AMF) form one of the most common symbiosis with the majority of land plants. AMF supply the plant with various mineral elements, primarily phosphorus, and improve the water supply. The search for the most effective AMF strains for symbiosis and the creation of microbial preparations on that basis is an important task for modern biology. Owing to the difficulties of cultivation without a host plant and their high genetic polymorphism, identifying AMF is very difficult. A high number of cryptic species often makes morphological identification unreliable. Recent years have seen a growth in the number of AMF biodiversity studies performed by modern NGS-based methods, Illumina MiSeq in particular. Currently, there are still many questions that remain for the identification of AМF. The most important are whether conservative or variable sequences should be used to select a marker for barcoding and whether universal primers or those specific to AMF should be used. In our work, we have successfully used universal primers ITS3 and ITS4 for the sequencing in Illumina MiSeq of the 5.8S rDNA – ITS2 region of the 35S rRNA genes, which contain both a conservative and variable regions. The molecular genetic approach for AMF identification was quite effective and allowed us to reliably identify eight of nine isolates to the species level: five isolates of Rhizophagus irregularis, and one isolate of R. invermaius, Paraglomus laccatum, and Claroideoglomus etunicatum, respectively. For all five R. irregularis isolates, high variability in the ITS region and the absence of ecotopic-related molecular characters in the ITS2 region were demonstrated. The NCBI data is still insufficient for accurate AMF identification of Acaulospora sp. isolates from the genus to the species level.

## Abstract (ru)

Грибы арбускулярной микоризы (АМГ) образуют один из наиболее распространенных симбиозов с большинством наземных растений. АМГ снабжают растение различными минеральными элементами, в первую очередь, фосфором, а также улучшают водоснабжение. Поиск наиболее симбиотически эффективных штаммов АМГ и создание на их основе микробных препаратов – важная задача современной биологии. Идентификация AМГ очень сложна. Это связано, прежде всего, с высоким генетическим полиморфизмом AMГ, а также с трудностями их выращивания без растения-хозяина. Морфологическая идентификация AMГ часто ненадежна из-за большого числа криптических видов. В последние годы увеличивается число работ по изучению биологического разнообразия AMГ, проводимых современными методами на основе NGS (Next Generation Sequencing), в частности Illumina MiSeq. В настоящее время остается много вопросов по идентификации АМГ. К наиболее важным из них относятся: выбор маркера для генетического штрих-кодирования AMГ – консервативных или вариабельных последовательностей, а также выбор праймеров – специфичных для AMГ или универсальных. В настоящей работе мы успешно использовали универсальные праймеры ITS3 и ITS4 для секвенирования с Illumina MiSeq региона 5.8S рДНК – ITS2, содержащего как консервативные, так и вариабельные участки. Этот подход для идентификации AMГ оказался достаточно эффективным и позволил достоверно идентифицировать 8 из 9 изолятов до уровня вида: 5 – Rhizophagus irregularis, 1 – R. invermaius, 1 – Paraglomus laccatum, 1 – Claroideoglomus etunicatum. Для всех изолятов R. irregularis показаны высокая вариабельность в области ITS и отсутствие связи c экотопом клад, образуемых ITS на филогенетических деревьях. Для изолята Acaulospora sp., определенного до рода, дан- ные NCBI все еще недостаточны для точной идентификации AMГ рода Acaulospora до вида.

## Introduction

Arbuscular mycorrhizal fungi (AMF) belonging to the subdivision Glomeromycotina are a relatively small yet diverse group. Various estimations report from 240 (Stockinger et al., 2014) to 348 species (Öpik et al., 2014). At the same time, AMF form mycorrhizal relationships with more than 200,000 species of land plants (Lee et al., 2013). Identification the AMF strains with the highest symbiosis efficiency is of great value for agricultural applications. To do it, we isolated, identified (Kryukov et al., 2017; Kryukov, Yurkov, 2018; Yurkov et al., 2018b), and evaluated the symbiotic efficiency (Yurkov et al., 2017a, b; Yurkov et al., 2018a) for a number of strains from the collection.

AMF are traditionally identified by more than 20 morphological characters (Schenk, Perez, 1990; Blaszkowski, 2019; INVAM, 2019; Schüßler, 2019). However, in some cases morphological identification fails to discriminate closely related species. A number of AMF species are morphologically indistinguishable. Furthermore, interspecific genetic polymorphism can sometimes be mistakenly treated as intraspecific polymorphism (Savary et al., 2017). Everything mentioned above may result in wrong estimations of AMF species. AMF usually cannot be cultivated on artificial media, thus making the identification very hard. As a result many AMF species are not studied at all, and so have not received the proper morphological description essential to their identification (Bruns et al., 2017). The type genus Glomus is a striking example of the taxonomic problems concerning species and genera identification. In the last twenty years, Glomus taxon has been revised a number of times, so that many Glomus species put under 14 other genera instead: Ambispora, Claroideoglomus, Corymbiglomus, Diversispora, Dominikia, Entrophospora, Funneliformis, Kamienskia, Pacispora, Paraglomus, Redeckera, Rhizophagus, Sclerocystis, Septoglomus (Schüßler, 2019). In this key, molecular-genetic identification of Glomeromycotina is very significant as the exponential increase in the number of AMF DNA sequences deposited in databases over the last ten years attests (NCBI, 2018).

The forementioned problems make the choice of the best method to identify Glomeromycotina genera and species very topical (Kryukov et al., 2017; Kryukov, Yurkov, 2018; Yurkov et al., 2018a).

The study of Glomeromycotina by molecular genetic methods is associated with a number of difficulties. One of the main problems is the high degree of genetic polymorphism, including polymorphism at the intraspecific level in the SSU–ITS1–5.8SrRNA–ITS2–LSU region commonly used for molecular-genetic identification (Stockinger et al., 2010; Yurkov et al., 2018a). The reasons for this variability may be: 1) the ability of AMF to form anastomoses and exchange genetic material (Daubois et al., 2016); 2) the formation of a very large number of nuclei – from 576 to 35,000 in one spore (Hosny et al., 1998). Copies of DNA markers independently evolve in different nuclei (Lin et al., 2014).

The ITS region is the main marker for AMF barcoding, it is well represented in different databases, among which are the UNITE (User-friendly Nordic ITS Ectomycorrhiza) (UNITE, 2017) and MaarjAM (Öpik et al., 2010). It should be noted that the ITS is also often used for the barcoding of vascular plants and the construction of phylogeny (Rodionov et al., 2016). But the main advantage of the ITS region is the possibility of identifying AMF up to the species level. A majority of papers where less variable markers have been used report on identification up to the genus level or even to the order level (Schoch et al., 2012; Tedersoo et al., 2015).

In the case of Illumina MiSeq it is recommended that ITS2 or the full ITS region be used as the fungal barcode (Tedersoo et al., 2015). ITS2 provides a higher taxonomic resolution than SSU or LSU genes, which are suitable for identifying genera and higher-level taxa. ITS1 in fungi is usually shorter that ITS2, also ITS1 is considered as a hypervariable region and thus less suitable for barcoding fungi (Tedersoo et al., 2015). To increase efficiency of molecular-genetic identification, modifications of the universal primers ITS3 and ITS4 specific for various fungal divisions were proposed. In comparison with other primer combinations, the primer pair ITS3tagmix and ITS4ngs gave a significantly larger number of sequencing reads as well as OTUs (Operational Taxonomic Units) (Tedersoo et al., 2015). At the same time, the contribution of AMF in the total OTUs pool was less than 3 %, whereas Agaricomycetes represented half of all OTUs. For AMF we proposed using the slightly modified primer ITS3 – CATC GATGAAGAACGTAG (the modification is in bold) as a direct primer and the primer ITS4 without changes as reverse.

Various aspects of using specific primers for the AMF identification were reviewed earlier (Kryukov et al., 2017; Kryukov, Yurkov, 2018; Yurkov et al., 2018a). Identification using universal primers allows us to investigate the maximal broad range of AMF species and genera. But this then introduces the problem of foreign DNA admixture. Another problem with molecular genetic identification of AMF is the generation of chimeric sequences during the sequencing process (Senés-Guerrero et al., 2014). Phusion DNA-polymerase, which generates high accuracy PCR-products, serves to reduce the chances of introducing chimera (Senés-Guerrero et al., 2014). At the same time, special software has been developed for detecting chimeric sequences and excluding them from analysis, USEARCH for example (Edgar, 2010).

Before 2015, the main method of AMF molecular-genetic identification was cloning followed by Sanger sequencing. This method applied to AMF demands careful selection of efficient and highly specific primers, as well as the use of nested PCR. In addition, due to the high variability of marker sequences, only multiple sequence cloning can be used (Krüger et al., 2009), which is very time-consuming and labor-intensive.

NGS has turned into a powerful and attractive method of AM fungi identification since it can overcome the weak points in the Sanger-based identification. One of the earliest techniques of NGS was 454 pyrosequencing, which has been employed for AMF identification since 2009. Using the universal fungal primers NS31 and AM1 179,279 sequences were obtained, of which 77.5 % belonged to 47 taxa of AMF isolated from the roots of 10 plant species (Öpik et al., 2009). However, these primers (NS31 and AM1) are not suitable for the analysis of the SSU region in Archaeosporaceae and Paraglomeraceae families (Helgason et al., 1998). Nonetheless, 454 pyrosequencing revealed in the roots of the Hepatica nobilis Mill. 1.5 times more fungal taxa than Sanger sequencing (Öpik et al., 2009). This clearly showed the advantage of NGS methods over cloning-Sanger sequencing methods. 454 pyrosequencing following nested PCR was successfully used for AMF identification with a barcode in the LSU region (Senés-Guerrero, Schüßler, 2015). An interesting result of this work is that about 60 % of the studied plants each formed a symbiosis with at least 10 AMF taxa, and 2 % of plants had more than 25 AMF species in their root system. The authors used the LSU-D1f modified primer (Senés-Guerrero, Schüßler, 2015) together with the LSUmBr primer (Krüger et al., 2009) in the second round of nested PCR. This modification allowed us to obtain 698,297 sequences, of which 0.17 % were the target AMF sequences, 41 taxa were detected, of which 15 are unknown, not registered in the databases (Senés-Guerrero, Schüßler, 2015). 454 pyrosequencing has its advantages over cloning-Sanger sequencing, but this technique is more expensive than the Illumina MiSeq technology that replaced pyrosequencing.

With the development of Illumina technology, Illumina MiSeq has been becoming more and more widely used due to its relative low cost. Illumina MiSeq compared to HiSeq 2000 allows for processing sequences of reads 2.5 times longer, and each sequencing in this case is cheaper. The advantage of HiSeq is more deep sequencing, which allows us to obtain reads at a rate of three orders greater than MiSeq. But this is less significant for fungi identification than sequence read length (Razzauti et al., 2015).

A comparative study of the efficiency of 454 pyrosequencing and Illumina MiSeq showed a difference of five times in the diversity of sequences (in favor of the second method), but both approaches revealed the same species composition (Vasar et al., 2017). A comparative study of the Illumina MiSeq and Ion Torrent Personal Genome Machine (PGM) showed that the second method generated a 2–5 fold greater rate of error than Illumina MiSeq (Salipante et al., 2014).

The objectives of this study included the identification of nine strains of AM fungi from the collection of the All Russian Research Institute for Agricultural Microbiology using the Illumina MiSeq approach and universal primers for the ITS2 region, supplemented by the morphological characteristics of the analyzed AMF spores.

## Materials and methods

AMF isolation. AMF were isolated in 2015 from samples, collected in various habitats in two different regions (author of the analyzed AMF isolates: A.P. Yurkov). Only isolates with spores with an unambiguously identifiable morphology were used for molecular-genetic identification. Four strains were isolated from samples taken in the Rostov region (1.4 km NW from Zernograd): 46°52ʹ2″ N, 40°16ʹ8″ E, a tree belt area with oaks, maples and alders: 1) isolate (strain) 01-053 was isolated from Ambrosia artemisiifolia roots, 2) isolate (strain) 01-056a and, 3) isolate 01-056b both were isolated from one ordinary chernozem soil sample, but differentiated via reinoculations of spores with a different morphology, 4) at 46°52ʹ7″ N, 40°16ʹ8″ E, a maize field, the isolate (strain) 02-060 was isolated from a Zea mays 282МВ Zelenogradckij hybrid roots. Five isolates were obtained from samples taken in the Moscow region (Lobnya town, academic village in Lugovaya): 1) 56°02ʹ33.80″ N, 37°29ʹ13.70″ E, natural meadow, the isolate (strain) 03-097 was isolated from Vicia sepium roots, 2) 56°02ʹ24.30″ N, 37°29ʹ20.00″ E, a Festuca rubra field, isolate (strain) 04-067 was isolated from Festuca rubra roots, 3) isolate (strain) 04-068 was isolated from Agrostis vulgaris roots, 4) 56°02ʹ31.40″ N, 37°29ʹ17.60″ E, Medicago × varia field, isolate (strain) 05-077 was isolated from Trifolium pratense roots, 5) isolate 05-104 was isolated from a sod-podsol gleyic soil sample.

AMF cultivation. AMF collection at the All-Russian Research Institute for Agricultural Microbiology is cultivated in the Plectranthus australis R. Br. (taxonomical synonyms P. verticillatus (L.f.) Druce, P. nummularius Briq.) line of Swedish ivy. For AMF-inoculated plants a soil-sand mix was used, described earlier (Yurkov et al., 2015). The substrate had a low level of plant-available phosphorus (3.0 mg P_2_0_5_/100 g). Plectranthus cuttings 12–15 cm length with two leaves were sterilized in 0.1 % sodium hypochlorite, then germinated in water. On the 7^th^ day Plectranthus plants were inoculated by root fragments, containing AMF vesicles and abruscules. Root fragments of mycorrhizal Plectranthus were selected by visual analysis in stereomicroscope MBS-10 (LZOS, Russia). For further reinoculations (each 6–8 months) sporocarps, or an arrangement of spores in an out-root area, or 5 mm length root fragments with large quantities of observable vesicles were used. Each inoculated plant was cultivated in an individual container with 350 g of sterile substrate at +24–26 °C, 18 h light day. Two luminescent lamp LB-40 (Russia) and OSRAM L36/77 Fluora (Germany) on a 1:1 ratio with output lumen ~4000 were used. Plants were watered every other day by 60 % of soil full water capacity. For the culture purification AMF spores were reinoculated at least three times.

Morphological identification of АМF. More that 20 features were used for morphological identification (Schenck, Pérez, 1990; Blaszkowski, 2003; INVAM, 2019): color, transparency, size and shape of extraradical (out-root) spores; shape of the place of attachment of spores to subtending hypha; number, thickness, density, elasticity or fragility, color in Melzer’s reagent of layers of spore walls and subtending hypha; presence/absence/disappearance/appearance of spore wall layers and subtending hypha during ontogenesis (from juvenile to mature spore); presence/absence of a septum in the place of attachment of the spores to subtending hypha; structural characteristics of AMF and intraradical spores.

In order to assess mycorrhization parameters and the type of mycorrhiza, the roots were macerated and stained by trypan blue according to the method developed by J.M. Phillips and D.S. Hayman (1970). Mycorrhization parameters were determined by light microscopy (Trouvelot et al., 1986) using a special computer program, developed earlier in our research group (Vorob’ev et al., 2016).

DNA extraction, PCR and sequencing. DNA extractions were carried out using the method of J.J. Doyle and J.L. Doyle (1987), with modifications. Micorrhizated roots of P. australis were washed twice in distilled water, placed in 2 ml tubes, dried at +45 °C, and mechanically homogenized with glass beads 2–4 mm in the FastPrep24 (MP Biomedicals, USA), followed by CTAB-protocol. The target region ITS2 was amplified with universal primers ITS3 (5ʹ-GCATCGATGAAGAACG CAGC-3ʹ) and ITS4 (5ʹ-TCCTCCGCTTATTGATATGC-3ʹ) (White et al., 1990). Ready-mix ScreenMix (Evrogen, Russia) was used for PCR. Amplicons were sliced from agarose gel and purified by the silica approach. Illumina library preparation was made according to MiSeq Reagent Kit Preparation Guide in the Core Center of “Genomic Technologies, Proteomics and Cell Biology” at the All-Russia Research Institute for Agricultural Microbiology (St. Petersburg, Russia). Libraries were sequenced on the Illumina MiSeq platform with MiSeq® Reagent Kit v3 (600 cycle) according to the manufacturer’s instruction (Illumina Inc., USA).

Bioinformatics and fungal OTUs analysis. Two pipelines were used for sequencing data analysis. 1. Illumina reads processing were done by USEARCH software (Edgar, 2010). The key steps for USEARCH data treatment are described on https://www.drive5.com/usearch/. In further paragraphs we are briefly describing these steps. In the case of paired-end sequencing, Illumina sequencer makes sequences from both ends of a fragment and generates two files with forward and reverse reads. This data is written in FASTQ format, where each nucleotide corresponds with its quality score. For further treatment forward and reverse reads are merging using fastq_mergepairs command to give consensus sequences. This step includes resolving any mismatches found in the overlap alignments and calculation the posterior quality scores for the consensus sequences (Edgar, Flyvbjerg, 2014). To discard low-quality reads expected error filtering with fastq_filter command is used.

The next step is a dereplication, which means sorting unique sequences in order of decreasing abundance in the dataset. After this, singletons (sequences that are present exactly once) are discarded, since they are likely to have errors. However the remaining reads can still have errors. So the goal of the final step (denoising) is to identify a set of correct biological sequences. The denoising can be made by UPARSE algorithm which clusters sequences with 97 % or more (Edgar, 2018) similarity and then chooses the most abundance sequence in each cluster. Also chimeric sequences, which occurs by combining parts of two or more biological sequences are detected and deleted at this step.

Raw forward and reverse reads were merged with minimal length parameter (“-fastq_minmergelen”) 130 bp and maximal difference parameter (“-fastq_maxdiffs”) 30 bp. Then lowquality read ends including primer sequences were trimmed, reads were filtered based on expected error value (E_max = 1). Singletons were removed from the dataset. Then data were divided on operational taxonomic units (OTUs) with a 97 % similarity cut-off by UPARSE algorithm (Edgar, 2013). Chimeric sequences were removed. For further analysis the most represented sequence from each OTU was chosen. Data were checked for cross-talk errors. Sequences of AMF species were selected by BLAST+ (Altschul et al., 1990).

2. The primers we used are universal for a broad range of species; and after amplification in the ITS2 region the extracted plant DNA prevail over fungi DNA. A second pipeline was made for the selection of rare and unique reads with a high homology of AMF sequences. After quality control (FastQC) forward and reverse reads were trimmed and merged with minimal length parameter 230 bp by trimmomatic (Bolger et al., 2014) and fastq-join software (Aronesty, 2013). Then sequences were demultiplicated and sorted in the descending order of their frequency. AMF sequences were selected via character for AMF motifs, then aligned and checked via BLAST.

Obtained sequences were submitted to the GenBank database (https://www.ncbi.nlm.nih.gov/). Evolutionary analyses were conducted by using the Maximum Likelihood method in MEGA7 software (Kumar et al., 2016) with implementation of the Tamura-Nei model (Tamura, Nei, 1993) and 1,000 bootstrap analyses.

## Results

**Fig. 1.**
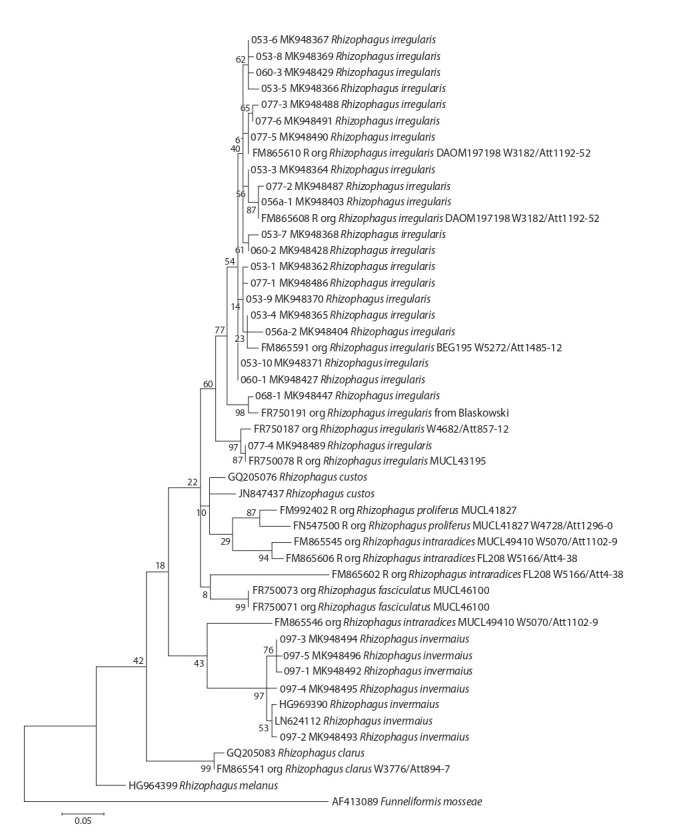
Phylogenetic tree of ITS-sequences from the genus Rhizophagus.

**Fig. 2.**
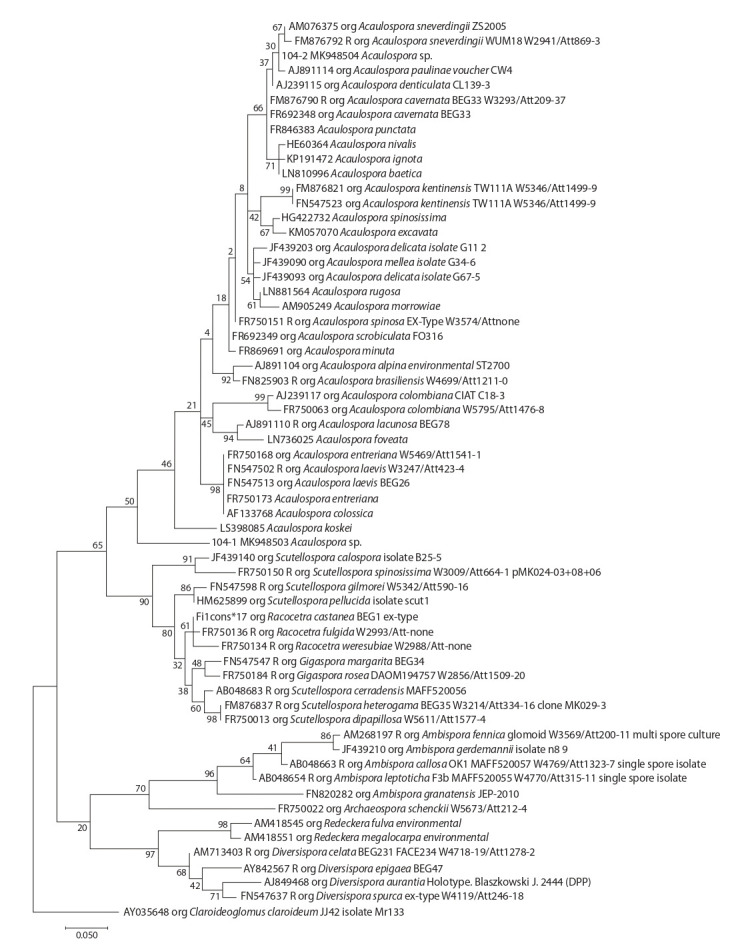
Common phylogenetic tree of ITS-sequences from Acaulospora, Archaeospora, Ambispora, Diversispora, Gigaspora, Racocetra, Redeckera and Scutellospora genera.

**Fig. 3.**
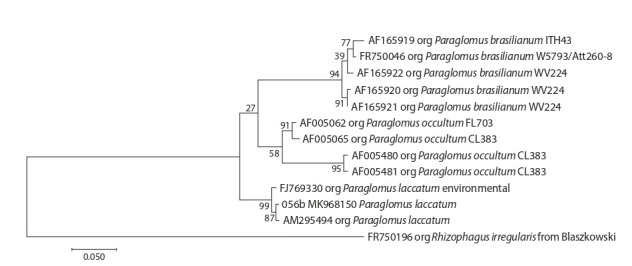
Phylogenetic tree of ITS-sequences from Paraglomus genus.

**Fig. 4.**
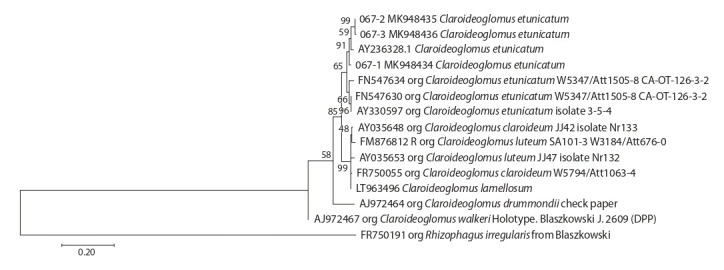
Phylogenetic tree of ITS-sequences from Claroideoglomus genus.

Sequencing of 9 isolates yielded approximately 381,249 pair of reads, from 19,236 to 81,054 joined sequences for each isolate. The following OTUs of AMF isolates were identified via BLAST at the genera or species level and submitted to NCBI: MK948362-MK948371 (isolate number 01-053), MK948403- MK948404 (01-056a), MK968150 (01-056b), MK948427- MK948429 (02-060), MK948492-MK948496 (03-097), MK948434-MK948436 (04-067), MK948447 (04-068), MK948486-MK948491 (05-077), MK948503-MK948504 (05-104). The length of obtained sequences varied from 340 to 366 bp. Variability in GC content was distinct in different genera: a narrow range was shown in genera Claroideoglomus (38–39 %), Rhizophagus (36–39 %) and Paraglomus (42–46 %), whereas GC content in Acaulospora varied from 31 to 46 %. Owing to the significant variability of the ITS region in AM, alignment of sequences belonging to different genera and orders is ineffective, and in some cases impossible. As a result, four separate phylogenetic trees were constructed for the four genera mentioned above (Fig. 1–4).

Unusual deletion was determined by ITS2 sequence alignment. This deletion of 5–6 bp in alignment positions 97–102 was identified in various species of genus Rhizophagus, and in all OTUs of genus Paraglomus, and in Racocetra weresubiae (FR750135) (NCBI, 2019). This fact can serve to indicate the presence of a deletion-specific site related to secondary RNA structure. However, it can also indicate a relationship of sequences in this region, which is of greater interest because it is well known that AM mycelia contain a significant number of nuclei (Hosny et al., 1998), some of which carry this deletion in ITS.

Some samples (01-053, 01-056a, 02-060, 04-068, 05-077, and 03-097) demonstrated a high similarity with Rhizophagus (see Fig. 1). OTU isolates 01-053, 01-056a, 02-060, 04-068, 05-077 fell with high accuracy into the clade formed by Rhizophagus irregularis, while OTUs of 03-097 were placed in one clade with R. invermaius (bootstrap index = 97). This genus includes a small number of species, about 20 according to A. Schüßler (2019), but in the NCBI only 8 species are represented by ITS sequences. Thus one may consider that Rhizophagus is a genus that requires further sequencing research. At the same time, it is beyond doubt that this study provides a molecular genetic identification at the species level of isolates 01-053, 01-056a, 02-060, 04-068, 05-077, 03-097.

Due to the ambiguous position of OTUs of isolate 05-104 on a pre-built phylogenetic tree of the Acaulospora genus, as well as due to the possibility that OTUs of this isolate could incorporate other close genera from the Acaulosporaceae family, species from the Archaeospora, Ambispora, Diversispora, Gigaspora, Racocetra, Redeckera and Scutellospora genera were added to the tree. We found that both OTUs of 05-104 isolates were included in the Acaulospora clade with high bootstrap support (see Fig. 2).

However, owing to the significant differences from other species of Acaulospora, we define the MK948503 sequence

(05-104 isolate) as a virtual taxon. The MK948504 sequence (05-104 isolate), though it has a high similarity with the A. sieverdingii and A. paulinae species, did not show similarities with the indicated species according to morphological data, therefore it was also identified as a virtual taxon. At the same time, it can be reliably stated that these two OTUs belong to different taxa, since they have significant differences in GC content (31 and 46 %). But differences did not appear in the case of morphological analysis, highlighting the obstacles of distinguishing among Acaulospora species.

The OTUs of 01-056b isolate formed a well-supported subclade in the Paraglomus genus (bootstrap index = 99) (see Fig. 3). Furthermore, the Paraglomerales order hosts two more genera (Innospora and Pervetustus) in addition to the Paraglomus genus, but the ITS sequences for them are unknown. Thus the outgroup for the phylogenetic tree was taken from another order. The 01-056b isolate was identified as Paraglomus laccatum. The sequences of the P. laccatum 01- 056b isolate turned out to be the shortest in comparison with isolates of other genera and ITS. Only three of eight species of this genera have ITS sequences in NCBI (Schüßler, 2019).

The Claroideoglomus genus is the most studied of the above mentioned genera. The ITS data were reported for six out of eight species (Schüßler, 2019). The OTU of isolate 04-067 with high support belongs to the clade formed by the species Claroideoglomus etunicatum (see Fig. 4).

To verify whether the molecular genetic identification of AMF was effective, we conducted a morphological identification of nine isolates (Supplementary)^1^. The principal morphological characteristics for comparison were: size, shape, and color of the spores in air by the CMYK standard; shape, thickness and color of spore layers in Melzer’s reagent by the CMYK standard; the shape and thickness of subtending hypha; the wall thickness of subtending hypha; the presence of a septum in subtending hypha, type of mycorrhiza in P. australis; mycorrhization parameter (F – frequency of mycorrhizal infection in P. australis roots).


Supplementary are available in the online version of the paper: http://www.bionet.nsc.ru/vogis/download/pict-2020-24/appx0.pdf



On the basis of morphological analysis of spores, it was concluded that the AM isolates under examination – supported on P. australis culture in the ARRIAM collection – are of the following taxa: 1) 01-053 isolate – Rhizophagus irregularis; 2) 01-056a – R. irregularis; 3) 01-056b – Paraglomus laccatum; 4) 02-060 – R. irregularis; 5) 03-097 – R. invermaius; 6) 04-067 – Claroideoglomus etunicatum; 7) 04-068 – R. irregularis; 8) 05-077 – R. irregularis; 9) 05-104 – Acaulospora sp. defined only at the genus level, since it was not possible to clearly determine distinguishing morphological features.

Among the morphological features of the spores formed by various isolates of AMF the following should be highlighted. The spores of all AMF isolates classified as R. irregularis (isolates 01-053, 01-056a, 02-060, 04-068 and 05-077) have the cylindrical subtending hypha in oblong spores or slightly flared (expanded) in spherical spore at a distance of more than 10 μm from the spores. The size, shape and color of the spores in air in these isolates were similar. Only spores of isolates 01- 053 and 01-056a with a greenish tint, had insignificant differences: color varied from 19-5-54-0 (pistachio) and 23-4-43- 0 (very light yellow-green) to 26-17-66-0 (yellow-green) and 29-25-58-0 (dark khaki). It should be noted that the color of the L3 layer in spores of R. irregularis isolates had significant polymorphism: from 10-37-99-10 (corn-yellow), 1-45-91-25 (deep yellow), 10-53-98- 0 (yellow-orange), 13- 54-99-1 (deep orange-yellow) and 26-52-97-2 (deep yellow) to 17-48-99- 2 (almost pure orange), 15-62-98-0 (deep orange-yellow), 2-67-82-58 (brown-red), 30-80-99-30, 32-78-99-25 (saturated red-brown). The spore color of the 01-056b isolate, referred to the Paraglomus laccatum species, on the contrary varied in a narrow range: from colorless to 4-4-0-0 (ghostly white). The second difference in the P. laccatum 01-056b isolate was the absence of a septum in the subtending hypha in the presence of narrowing at the site of attachment of the spore as determined by the greater thickness of the L2 layer. The spores of isolate 04-067, referred to Claroideoglomus etunicatum, were more yellow: from 5-5-0-0 (ghostly white) to 2-10-62- 5 (orangeyellow Craiol) in air and in Melzer’s reagent L2 had a yellow color from 2-15-60-2 (orange-yellow Craiol) to 3-35-92-5 (saturated yellow). The darkest spores were observed in the isolate 03-097, referred to the Rhizophagus invermaius species: from 30-52-88-51 (deep yellow-brown) to 59-67-63-72 (brown-olive) in air. The spore morphology of isolate 05-104 was significantly different from the other AMF. The spore color of isolate 05-104 attributed to the Acaulospora genus, ranged from colorless (transparent) to 3-15-70-4 (yellow ivory), spores cycatrix (the scarring is a remnant of the connection between the spore wall and the wall of the spore of the saccule during spore synthesis is 5.5–7.1 μm. Since this isolate is poorly maintained in P. australis culture, the morphology of the spores could not be studied in detail. According to molecular genetic analysis data, it may be approximately the same probability as A. paulinae, A. denticulata or A. sieverdingii.

All isolates of the Rhizophagus genus (01-053, 01-056a, 02-060, 03-097, 04-068 and 05-077), collected both in the forest belt and arable land, had a higher activity in P. australis roots (F > 80 %) than isolates of other AMF genera (see Supplementary). All the AMF isolates under examination formed mycorrhiza of the Arum-type, but only seven of nine isolates were able to be isolated into culture: 01-053, 01-056a, 02-060, 03-097, 04-067, 04-068, 05-077 isolates should be considered as AMF strains.

Because their mycorrhization activity in P. australis is low (see Supplementary), sustained maintenance of the P. laccatum 01-056b isolate and the Acaulospora sp. 05-104 isolate will depend on the selection of optimal conditions for their growth (substrate composition and type of host plant).

## Discussion

The Illumina MiSeq approach allowed us to determine eight out of nine AMF isolates from the All Russian Research Institute for Agricultural Microbiology collection. It is very powerful method, which enables the identification of a large number of AMF taxa in fungal communities, especially if the proportion of targeted marker sequences in a sample is small. There are other effective methods of NGS, but several comparative studies indicate that MiSeq in some cases is more efficient as it can provide longer reads and fewer errors in comparison to HiSeq and IonTorrent techniques, respectively (Salipante et al., 2014; Razzauti et al., 2015).

These comparisons show the clear advantage of the sequence- targeted NGS approach as contrasted with the alternative cloning-sequencing method for AMF species identification. However, the main disadvantage of the Illumina MiSeq are relatively short reads (250 bp ×2), which do not allow the use of long markers, such as the entire cloned SSU-ITS1-5.8SITS2- LSU region that was most often used for AMF barcoding from 2009 to 2012 (Krüger et al., 2009; De Castro et al., 2018). A prerequisite for the correct AMF identification by the MiSeq method is the employment of a short marker region for sequencing (400–500 bp). The most commonly used lengthappropriate marker is ITS2. The advantage of this region is that it provides sufficient variability for identification at the species level. Other less variable regions such as D1–D2 of the LSU allow identification only at the genus level (Krüger et al., 2009).

The use of universal primers for ITS2 region is the optimal choice for the identification of poorly studied taxa, which in recent years has helped to identify a significant number of virtual taxa of AMF (Öpik et al., 2014). These taxa can subsequently receive species names in the presence of individual morphological features and stable maintenance of isolates/strains in culture. For example, we studied the 05- 104 sample in which there are two OTUs that belong to two different virtual Acaulospora sp. taxa. Another advantage of using the ITS2 region for identification is that a substantially representative sequence database has been stored in NCBI in comparison to other marker regions, for example, for the SSU region (NCBI, 2018).

It is important to note that OTUs related to one R. irregularis species and collected in different ecotopes (isolates 01-053, 01-056a, 02-060, 04-068 and 05-077) did not cluster separately on the phylogenetic tree (see Fig. 1). This suggests that all studied R. irregularis isolates shared one ribotype. Thus, it was not possible to identify the ecotype-related features of R. irregularis isolates, perhaps they are objectively missing.

Also, it should be noted that NGS methods produce huge data arrays. We paid special attention to choose the correct tools for data treatment. Several pipelines have been developed to process rDNA sequences that are generated using Illumina’s MiSeq platform. Among them mothur (Schloss et al., 2016), QIIME (Caporaso et al., 2010), USEARCH and VSEARCH (Rognes et al., 2016) are the most popular. Generally, all of them have similar steps in sequences treatment. We used USEARCH since it has a large set of tools and detailed documentation. However, two approaches were used to search for AMF OTUs among a great pool of sequences. Both approaches were equally effective since they allowed us to identify the same set of taxa. At the same time the use of the USEARCH software has significantly reduced the time spent on data processing, which makes it possible to recommend it as the main tool to identify AMF from NGS data.

Combining the results of molecular genetics and morphological identification, we assert that the effectiveness of the Illumina MiSeq method as applied to AMF identification is not inferior to morphological methods that are significantly more labor intensive. However, the NCBI database is still insufficient for identification of some AMF species. The reason for this is that more than half of the known AMF taxa are still absent in the database. For example, according to A. Schüßler (2019), there are up to 56 species in the genus Acaulospora. The NCBI contains 37 species just for Acaulospora sp., and ITS sequences are presented for only 28 species. Thus, the obtained OTUs of the 05-104 isolate may belong to one of Acaulospora species that has yet to be studied.

## Conclusion

We have determined that the most effective method for AMF identification is Illumina MiSeq supplemented by application of universal primers for the ITS2 region. Considerable efforts of morphologists in collaboration with molecular geneticists are required to establish a reliable taxonomy of the Glomeromycotina subdivision and to improve the efficiency of the molecular genetic AMF identification as a key method in the future.

## Conflict of interest

The authors declare no conflict of interest.
